# Factorial Optimization and Validation of Solid–Liquid Extraction with Low-Temperature Partitioning of Pesticide Residues from Pequi Pulp Using High-Performance Liquid Chromatography with a Photodiode Array Detector (HPLC-DAD)

**DOI:** 10.1021/acsomega.5c01790

**Published:** 2025-04-22

**Authors:** Marcelo O. Ferreira, João Pedro M. Silva, Mara R. R. Pereira, Allyson L. R. Santos, Anizio M. Faria

**Affiliations:** Institute of Exact and Natural Sciences of Pontal, Federal University of Uberlandia, Ituiutaba 38304-402, Brazil

## Abstract

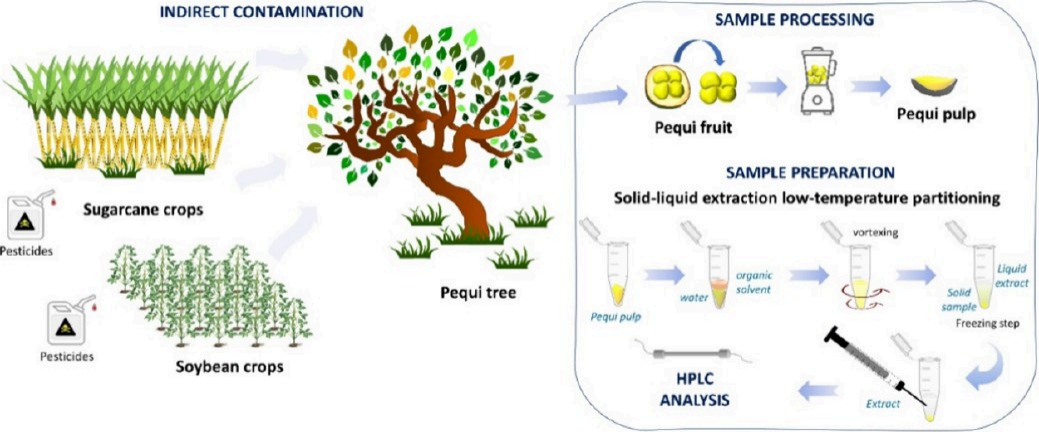

In this study, we developed a solid–liquid extraction method with low-temperature partitioning for extracting six pesticide residues (carbendazim, diuron, carboxin, chlorpyrifos, terbutylazine, and thiabendazole) from pequi pulp. Pequi is a key fruit of the Brazilian Cerrado biome, which is characterized by its intensive agricultural activity. This sample preparation technique was chosen for its simplicity, minimal analyst intervention, and effective extraction of pesticide residues from food samples. The selected pesticides reflect those widely used in grain and sugar cane monocultures within the Cerrado biome. The optimization of pesticide extraction from pequi pulp samples was conducted by using experimental design approaches. Pesticide recoveries ranged from 86 to 118% under optimized conditions, which included 500 mg of sample, 7.2 mL of ethyl acetate, 3.4 mL of water, and a freezing time of 3 h. The validated method, performed in a single step without additional cleanup procedures, demonstrated selectivity, robustness, and limits of quantification below 6.9 μg kg^–1^, as well as high efficiency. No pesticide residues were detected in the pequi samples collected from strategic cities in the Cerrado. This absence may be attributed to the protective nature of the thick external epicarp, which shields the pulp from pesticide contamination, even in areas with intensive agricultural activity.

## 1 Introduction

The Cerrado is the second-largest Brazilian biome, responsible for almost 25% of the territory, and has rich and complex biodiversity. It is estimated that the Cerrado has more than 1000 species of trees, among which fruit trees occupy a prominent place in the ecosystem’s richness.^1^ The pequizeiro (*Caryocar brasiliense* camb.) can be considered to be the symbolic tree of the Cerrado. Its fruit, pequi, is the best-known and most consumed in the region. Pequi has a thick green-brown epicarp, a white external mesocarp, and a yellow internal mesocarp (pulp) coated by a spiny endocarp, which protects the seed in the central part of the fruit. Pequi pulp, the edible part of the fruit, is rich in lipids (60% unsaturated fatty acids), fiber, carotenoids (responsible for its yellow color), phenolic compounds (a potential source of antioxidants), and minerals.^2−6^ These polyfunctional characteristics associated with its sui generis flavor have expanded commercialization and consumption beyond the Brazilian market.^7^

On the other hand, Cerrado is the main production area of Brazilian agribusiness, whose model is based on the intensive use of pesticides and fertilizers.^8^ Expanding agricultural activities, especially in soybean and sugar cane cultures, have decreased biome biodiversity, led to environmental degradation, and resulted in pesticide contamination of protected areas and local communities.^8−10^ Cerrado fruit trees and their fruits can be at risk of indirect contamination by pesticides applied to the monocultures. However, to the best of our knowledge, no method to determine pesticide residues in Cerrado fruits - baru (*Dipteryx alata*), araticum (*Annona montana*), buriti (*Mauritia flexuosa*), murici (*Byrsonima crassifolia*), mangaba (*Hancornia speciosa*), bocaiuva (*Acrocomia aculeata*), especially in pequi, has been reported in the literature or by the Pesticide Residue Analysis in Food Program (PARA, Programa de Análise de Resíduos de Agrotóxicos em Alimentos) from Brazilian health surveillance agency.^11^

The QuEChERS (quick, easy, cheap, effective, rugged, and safe) method has guided the extraction of pesticide residues from food matrices in the last two decades.^12−14^ The QuEChERS method and its modifications have successfully extracted different pesticide molecules from several food matrices.^14−19^ Solid–liquid extraction with low-temperature partitioning (SLE-LTP) has also efficiently extracted pesticide residues from fruits and vegetables using reduced procedures with minimal analyst manipulation.^20−23^ SLE uses a water-miscible, less-dense organic solvent as an extractor. The pesticide molecule partition between the sample and organic solvent begins at room temperature and is completed during the freezing step. A water volume is required to facilitate the partitioning process and maintain the sample matrix at a frozen state.^5,20,21,23,25^ After freezing, the organic phase containing the pesticides remains liquid and can be directly analyzed in a chromatographic system without an additional cleanup step. Therefore, the entire extraction process can be conducted in a single pot.^21,25,26^

This work proposes an analytical method for determining pesticide residues in pequi pulp by high-performance liquid chromatography with a photodiode array detector (HPLC-PDA). Due to the complexity of the pequi matrix and the need for simple methods that any analytical laboratory can use to monitor the proposed method, it was based on solid–liquid extraction with low-temperature partitioning. Six pesticides (carbendazim, thiabendazole, carboxin, diuron, terbuthylazine, and chlorpyrifos), chosen based on the frequency of use in the past decade in Cerrado monocultures, were extracted from pequi pulp by an optimized and validated SLE-LTP method without additional cleanup steps, following the method validation guidelines for pesticide residue analysis in food.^27^

## 2 Methods

### 2.1 Materials

Methanol and acetonitrile of HPLC-grade were used as the mobile phase and purchased from JT Baker (Xalostoc, Mexico). Ethyl acetate, 99%, was used as the extraction solvent and purchased from Ecibra (Santo Amaro, Brazil). Deionized water was ultrapurified, with a resistivity greater than 18.3 MΩ cm, in a MegaPurity Mega UP system (Billerica, MA, USA). All solvents were filtered through 0.22 μm poly(tetrafluoroethylene) (PTFE) membranes (Millipore, Bedford, MA, USA). Carbendazim (CBDZ) (97%), carboxin (CARB) (99%), chlorpyriphos (CHLO) (99%), diuron (DIUR) (98%), terbuthylazine (TERB) (99%), and thiabendazole (THIA) (98%) were analytical standards obtained from Pestanal (Rieder-del-Haen, PA, USA). The physicochemical properties of the pesticides are presented in Table 1.

**Table 1:**
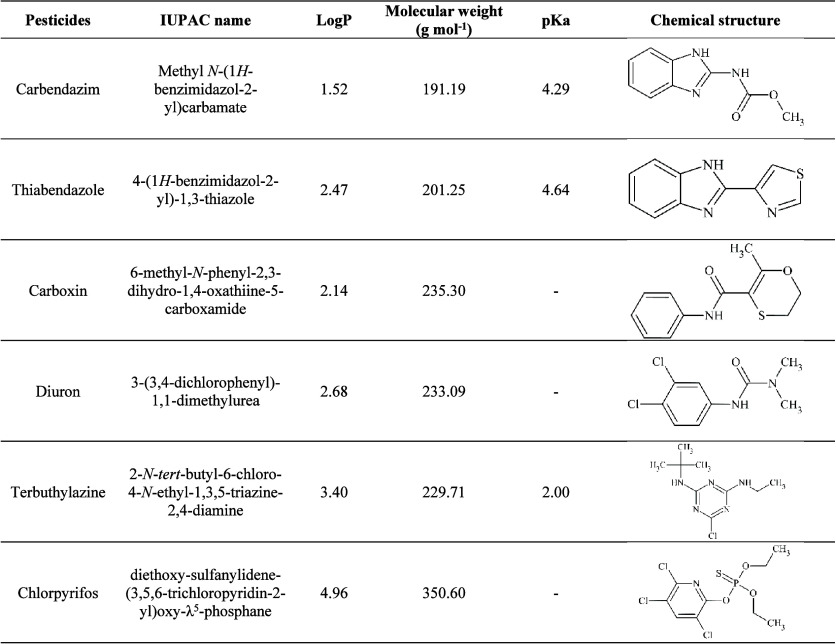
Physicochemical Properties of the Studied Pesticides in the Pequi Pulp Samples

### 2.2 Preparation of Solution and Pequi Pulp Test Portions

Stock solutions of a mixture of pesticides (CBDZ, CARB, CHLO, DIUR, TERB, and THIA) were prepared in methanol at 1000 μg mL^–1^ and stored in a freezer at −4 °C. Working standard solutions were prepared by diluting stock solutions with methanol.

The pequi fruits evaluated in this work were acquired from the Central de Abastecimento de Minas Gerais S.A. (CEASA-MG) in Uberlândia, Minas Gerais, Brazil. The pequi fruits were washed with water; the pequi shell was removed, and their pulp was cut into small pieces. Pequi pulp was homogenized for 5 min at room temperature using a multiprocessor (MALLORY, Oggi Black). Then, the pequi test portions were stored at −20 °C in a freezer until they were used.

### 2.3 Optimization of Solid–Liquid Extraction with Low-Temperature Partitioning

Before optimizing the SLE-LTP of pequi pulp pesticide residues, a preliminary study using a fractional factorial design (FFD) was conducted to evaluate the variables that most significantly affect the extraction efficiency. Extractor solvent, extractor volume, freezing time, sample mass, water volume, use of centrifugation, type of stirring, and contact time between pequi pulp and pesticide standards were the variables evaluated in a 2^8-4^ fractional factorial design. The variables and levels studied are presented in Table 2, and the experimental matrix for FFD 2^8-4^ is presented in Table 3.

**Table 2:** Factors and Levels Studied in a 2^8-4^ Fractional Factorial Design for ESL-PBT of Pesticides in Pequi Pulp

variables		level (−)	level (+)
A	solvent type	acetonitrile	ethyl acetate
B	solvent volume/(mL)	4.0	8.0
C	freezing time/(h)	3	12
D	sample mass/(g)	0.5000	1.000
E	water volume/(mL)	2.0	4.0
F	use of centrifugation	no	yes
G	stirring type	vortex	ultrasound
H	contact time (pequi pulp/pesticide sol.)/(min)	15	30

**Table 3:** Experimental Matrix for the 2^8-4^ Fractional Factorial Design for Extracting Pesticides from Pequi Pulp^a^

Exp.	A	B	C	D	E	F	G	H
1	–	–	–	–	–	–	+	–
2	+	–	–	–	+	–	–	+
3	–	+	–	–	+	+	–	–
4	+	+	–	–	–	+	+	+
5	–	–	+	–	+	+	–	+
6	+	–	+	–	–	+	+	–
7	–	+	+	–	–	–	+	+
8	+	+	+	–	+	–	–	–
9	–	–	–	+	–	+	–	+
10	+	–	–	+	+	+	+	–
11	–	+	–	+	+	–	+	+
12	+	+	–	+	–	–	–	–
13	–	–	+	+	+	–	+	–
14	+	–	+	+	–	–	–	+
15	–	+	+	+	–	+	–	–
16	+	+	+	+	+	+	+	+

a Variables E = A × B × C, F = B × C × D, G = A × B × C × D, H = A × C × D.

Six factors were fixed after this study. The factors with the most significant effects on pesticide extraction from pequi pulp using the SLE-LTP technique—water volume and extractor solvent—were optimized by a central composite design (CCD).

Pequi pulp samples were spiked with a standard pesticide mixture, obtaining a final concentration of 0.100 mg kg^–1^ of each pesticide. The homogenization of the spiked pulp was obtained by vortexing for 1 min. After 15 min at room temperature, 0.5 g of spiked pequi pulp was placed in a 50 mL Falcon tube, and water and ethyl acetate were added to perform 11 (11) extractions in combinations of different volumes of these solvents, as shown in Table 4. Then, the Falcon tube was vortexed for 1 min and subsequently frozen at −4 °C. After three h, the lower aqueous phase was transferred into a Falcon tube and frozen. An aliquot of 4 mL of the upper organic phase was collected. The ethyl acetate was evaporated from 4 mL of the extract and resuspended in 0.2 mL of acetonitrile. The resuspended extract was injected into the HPLC system.

**Table 4:** Combinations of Water and Extractor Solvent Volumes for Optimizing the SLE-LTP Method of Pesticides in Pequi Pulp as Determined by a Central Composite Design

Exp.	volume of ethyl acetate (mL)	volume of water (mL)
1	6.0 (−)^a^	1.0 (−)
2	10.0 (+)	1.0 (−)
3	6.0 (−)	3.0 (+)
4	10.0 (+)	3.0 (+)
5	8.0 (0)	2.0 (0)
6	8.0 (0)	2.0 (0)
7	8.0 (0)	2.0 (0)
8	5.2 (−1.4)	2.0 (0)
9	10.8 (+1.4)	2.0 (0)
10	8.0 (0)	0.6 (−1.4)
11	8.0 (0)	3.4 (+1.4)

a (coded values).

### 2.4 Method Validation

The optimized method was validated by the guidelines for method validation procedures for pesticide residue analysis in food, as outlined in the SANTE/11312/2021 document.^27^ The procedure subjected to method validation consisted of adding 0.5 g of spiked pequi pulp, 3.4 mL of water, and 7.2 mL of ethyl acetate to a 50 mL Falcon tube. Then, the Falcon tube was vortexed for 1 min and frozen at −4 °C for 3 h. After that, an aliquot of 4 mL of the upper organic phase was collected. The ethyl acetate was entirely evaporated under a flow of argon and then resuspended in 0.2 mL of acetonitrile. The resuspended extract was injected into the HPLC system. The SLE-LTP was validated in selectivity, matrix effect, range, linearity, limit of quantification (LOQ), precision (repeatability and within-laboratory reproducibility), and recovery.

The pequi pulp test portion was fortified with the stock standard solution and was used in the validation study. Linearity and range were determined by analyzing both matrix-matched and solvent pesticide standards in triplicate. The matrix effect was calculated using eq 1 by comparing the responses from solvent and matrix-matched standards.
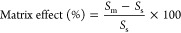
1Where *S*_m_ is the slope of the analytical curve obtained using matrix-matched standards, and *S*_s_ is the slope of the analytical curve obtained using solvent standards.

Spiked test portions at two different concentrations were analyzed in 6 replicates on three different days to evaluate the repeatability (intraday precision), within-lab reproducibility (interday precision), and accuracy of the SLE-LTP method. The accuracy of the SLE-LTP method was determined by the mean recoveries of pesticide residues in spiked samples (eq 2). Precision was assessed as the relative standard deviation (RSD) of the replicated intraday and interday experiments.
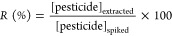
2Where *R* (%) = recovery percentage, [pesticide]_extracted_ = pesticide concentration obtained by the SLE-LTP method, and [pesticide]_spiked_ = pesticide concentration spiked in the pequi pulp sample.

The limits of quantification (LOQ) were evaluated as signal-to-noise ratios of 10:1. The pesticide concentration that led to a signal ten times the noise level was assessed using the average of the peak areas of the spiked samples in triplicate and considering the values of the noise level (measured in the chromatogram of a blank sample).

The acceptability criteria for the proposed method were defined according to the SANTE/11312/2021 document,^27^ in which the mean recoveries of pesticides should be within the range 70–120%, with an associated repeatability RSD ≤ 20% for all compounds; the linearity of the method should have a coefficient of correlation (*r*) > 0.99, and the matrix effect ≤ 20% for each pesticide.

### 2.5 Application of the SLE-LTP Method

The proposed SLE-LTP method was applied to samples of pequi pulps obtained from the local commerce in different regions of the Brazilian Cerrado. Samples were obtained from the cities of Santa Terezinha de Goiás (sample no. 1), Quirinópolis (samples #2 and #3), Iporá (sample no. 4), and Porangatu (sample no. 5) in the state of Goiás. Each sample represented a batch of approximately 1 kg of raw samples, with each fruit weighing between 15 and 20 g of pulp. The optimized and validated SLE-LTP method was used to extract the samples in triplicate. Additionally, these samples were fortified with a standard solution of the pesticide mixture at 10 μg kg^–1^ to assess the effectiveness of the proposed method for real samples.

### 2.6 Chromatographic Analysis

The pequi pulp extracts obtained using the SLE-LTP method were analyzed with a Waters Alliance e2695 HPLC system (Milford, MA, USA) equipped with a quaternary pump, autosampler, column oven, and photodiode array detector (PDA). In each chromatographic analysis, 10 μL of pequi pulp extracts were injected. A reversed-phase XBridge C_18_ (4.6 × 150 mm, Waters) column was used for chromatography. A mobile phase consisting of water (A) and MeOH (B) was used at a flow rate of 1.0 mL min^–1^. The gradient elution started at 50% mobile phase A for 3 min, with a linear decrease to 30% mobile phase A in 7 min and 0% mobile phase A in 8 min, followed by isocratic elution for 12 min with a linear increase to 50% mobile phase A by 13 min. The total time for each chromatographic run was 13 min. The column temperature was 25 °C, and the analyte wavelengths used were 230, 254, and 285 nm. Data were acquired and processed using Empower3 software.

## 3 Results and Discussion

### 3.1 Optimization of the SLE-LTP Method

The SLE-LTP method involves the solvent extraction of pesticide residues from food matrices. However, unlike traditional solvent extraction methods, the separation of the extract from the sample matrix occurs due to the freezing of the aqueous phase, maintaining the organic extract liquid. In this method, the extractor solvents are miscible in water, providing several advantages, including lower toxicity of the extractor compared to traditional solvent extraction techniques, more extensive contact between the matrix sample and the extractor, and reduced sample handling, which reduces both time and sources of error.^25^ A potential advantage is the possibility of carrying out the steps of the extraction process in one pot (Falcon tube) without requiring additional cleanup steps or transfer to other flasks.

**Figure 1:**
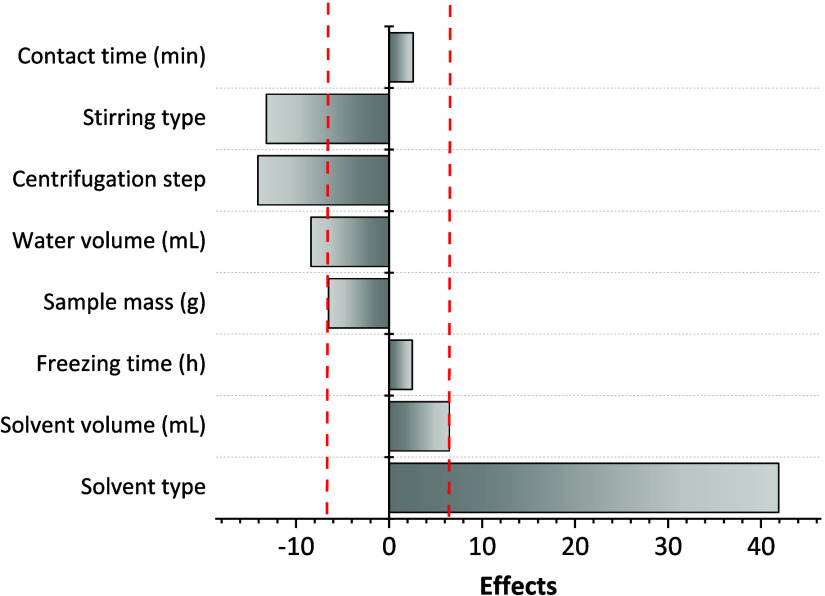
Pareto chart for 2^8-4^ fractional factorial design of low-temperature partitioned solid-phase extraction of pesticides from pequi pulp.

Initially, a 2^8-4^ fractional factorial design (FFD) was employed to identify the factors influencing the extraction of pesticide residues from the pequi pulp. This study evaluated eight factors: extractor type and volume, freezing time, sample size, water volume, centrifugation step, stirring type, and contact time between the sample matrix and pesticide standards. These factors were selected based on the SLE-LTP method applied to other food matrices.^21,24,28^ In this preliminary study, six factors were fixed due to their low influence on pesticide extraction or because they were categorical variables (Table 5 and Figure 1). After the FFD, the variables optimized for higher pesticide recoveries were a test portion mass of 500 mg, a contact time of 15 min, a freezing time of 3 h without a centrifugation step, vortex stirring, and ethyl acetate as the extraction solvent. Beyond its higher efficiency compared to acetonitrile, ethyl acetate enables the one-pot partitioning of pesticides between aqueous and organic phases.^29,30^ The non-fixed factors, including volumes of water and ethyl acetate, were optimized using a central composite design (CCD) for pesticide extraction from pequi pulp by the SLE-LTP method.

**Table 5:** Results from a 2^8-4^ Fractional Factorial Design Study on Pesticide Recoveries (%) from Pequi Pulp Using the SLE-LTP Method (*n* = 3)

	pesticide recoveries (%)					
Exp	carbendazim^a^	thiabendazole^a^	carboxine^b^	diuron^b^	terbuthylazine^c^	chlorpyrifos^c^
1	51 ± 1	54 ± 3	46 ± 3	48 ± 2	25 ± 4	4 ± 3
2	77 ± 2	86 ± 6	101.3 ± 1.1	102 ± 1	98 ± 2	114 ± 2
3	58 ± 2	58 ± 5	54 ± 3	55 ± 3	30 ± 2	2 ± 1
4	75 ± 10	81 ± 9	82 ± 9	85 ± 9	77 ± 7	82 ± 4
5	45 ± 2	47 ± 1	41 ± 1	41 ± 1	17 ± 1	8 ± 1
6	72 ± 9	75 ± 9	79 ± 10	80 ± 9	75 ± 10	84 ± 10
7	65 ± 1	66 ± 7	61 ± 4	62 ± 4	39 ± 5	2 ± 1
8	80 ± 4	85 ± 4	94 ± 2	95 ± 2	88 ± 1	108 ± 3
9	49 ± 3	52 ± 2	38 ± 2	40 ± 3	14 ± 1	7 ± 5
10	54 ± 6	49 ± 5	47 ± 6	49 ± 6	43 ± 6	49 ± 7
11	55 ± 2	54 ± 3	49 ± 2	50 ± 2	24 ± 1	2 ± 1
12	86 ± 1	88 ± 1	92 ± 1	92 ± 1	89 ± 5	118 ± 16
13	45 ± 4	44 ± 4	39 ± 4	40 ± 4	20 ± 4	12 ± 1
14	81 ± 12	90 ± 8	96 ± 7	96 ± 7	94 ± 5	128 ± 5
15	67 ± 1	68 ± 2	59 ± 2	59 ± 2	25 ± 2	2 ± 1
16	58 ± 3	65 ± 4	62 ± 2	64 ± 2	53 ± 1	82 ± 3

a Detection at 285 nm.

b Detection at 254 nm.

c Detection at 230 nm.

Eleven SLE-LTP experiments combining different water and ethyl acetate volumes were performed, as indicated by the central composite design (CCD) in Table 4. The mean recoveries (%) of the six pesticides from the pequi pulp test portions, calculated using eq 2, are presented in Table 6.

**Table 6:** Mean Recoveries of Pesticides Extracted from Pequi Pulp, Fortified at 0.1 mg kg^–1^, Using the SLE-LTP Method, by the CCD Experiments (*n* = 3)

	recovery (%)					
Exp.	CBDZ	THIA	CARB	DIUR	TERB	CHLO
1	90	87	88	91	87	101
2	96	96	97	100	89	97
3	92	94	98	102	93	102
4	94	91	95	101	90	91
5	90	94	98	104	96	98
6	92	91	96	101	91	95
7	93	93	97	103	95	97
8	85	90	97	102	96	103
9	87	84	83	88	76	71
10	90	94	92	98	88	90
11	88	89	98	103	97	104

**Figure 2:**
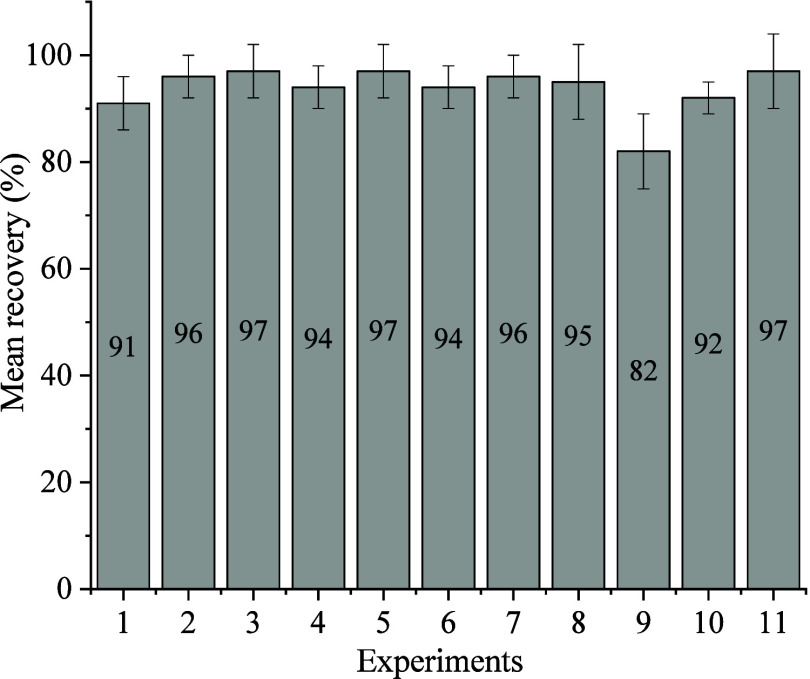
Average recoveries of the six pesticides from pequi pulp, fortified with 0.1 mg kg^–1^, by the SLE-LTP method according to the central composite design experiments (*n* = 3).

The mean recovery of pesticides was 71 to 104%, meeting the acceptability criterion for pesticide recoveries in food matrices (70–120%) regardless of water and ethyl acetate volumes. The relative standard deviation (RSD) obtained from the pesticide recoveries at the center point of the CCD experiments was 1.3%, resulting in a high precision of the experimental design. The average recoveries of the six pesticides in each CCD experiment were used to optimize the extractor and water volumes for simultaneous pesticide extraction. Figure 2 presents the average recoveries of six pesticides from the pequi pulp for each CCD experiment.

The average recoveries of the six pesticides in each experiment varied from 82 to 97%. The experiment (no. 9) that resulted in the lowest pesticide recovery used the larger extractor volume. The average pesticide recoveries in each CCD experiment (Figure 2) were used to determine the optimal volumes of water and ethyl acetate in the SLE-LTP. Fang et al. also used the average pesticide recoveries as a response factor for extraction optimization via experimental design.^31^ The model equation used to obtain the predicted average pesticide recoveries is given in eq 3.
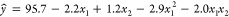
3Where  is the predicted average recovery of pesticides from pequi pulp samples, *x*_1_ and *x*_2_ represent the linear effects of ethyl acetate and water volumes, respectively.

Table 7 presents the results of validating the model’s coefficients using Analysis of Variance (ANOVA) in Microsoft Excel.

**Table 7:** Analysis of Variance for the Regression Model of Pesticide Recovery from Pequi Pulp Using the SLE-LTP Method

source	sum of squares	degrees of freedom	mean square	*F*-test
regression	120.23	5	24.05	
residual	75.30	5	15.06	
lack of fit	71.99	3	24.00	14.51
pure error	3.31	2	1.65	
total	196.06	10		
*R*^2^	98.26			
adjusted *R*^2^	61.49			

**Figure 3:**
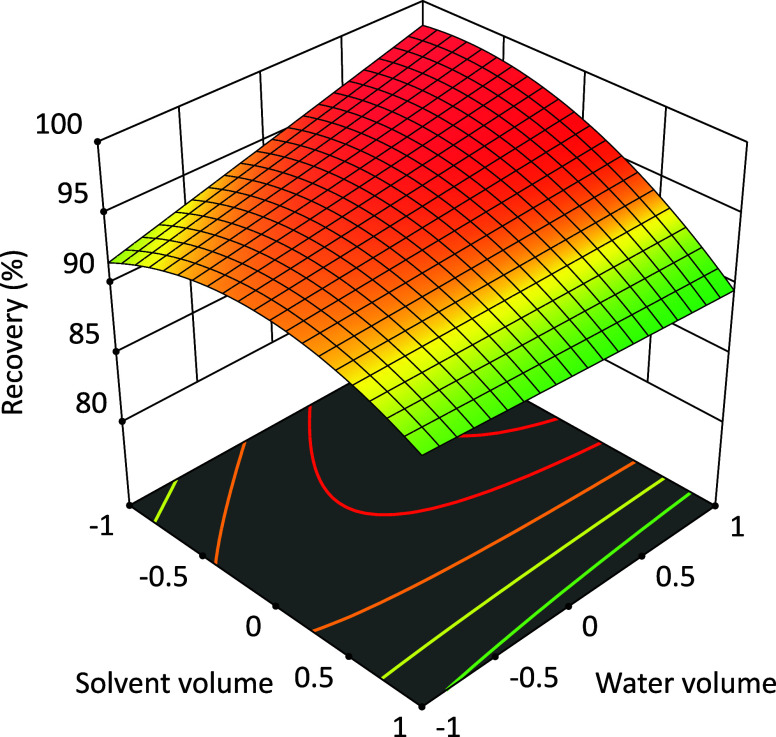
Response surface methodology for the average recoveries of six pesticides from pequi pulp using the SLE-LTP method.

The regression and adjusted regression coefficients (*R*^2^, adjusted *R*^2^) and the *F*-test evaluated the model’s lack of fit. In both cases, the model did not exhibit a significant lack of fit, despite having a low adjusted *R*^2^ value. This low value for the adjusted *R*^2^ may be related to combining individual pesticide recoveries in each experiment to predict their optimal simultaneous extractions in the CCD. Thus, the optimal extraction conditions for the pesticides from Pequi pulp were visually determined using the response surface methodology (Figure 3), as also reported by other authors for optimizing the multiresidue extraction of pesticides.^32−35^ The best condition for simultaneous pesticide extraction from the pequi pulp requires 7.2 mL (*x*_1_ = −0.4) of ethyl acetate and 3.4 mL (*x*_2_ = +1.4) of water. Under these conditions, the model predicts an average pesticide recovery of 99%. According to the response surface methodology in Figure 3, the maximum average pesticide recovery occurred outside the experimental conditions tested. The maximum recovery is obtained as the water volume increases above 3.4 mL. On the other hand, the maximum recovery predicted by the model is close to 100%, which aligns with the acceptance criteria for pesticide residue recovery (70–120%).^27^ The water volume used was sufficient to freeze all pequi pulp matrices. Thus, the SLE-LTP method was considered to be optimized under the previously cited conditions.

### 3.2 Analytical Performance of the SLE-LTP Method

**Figure 4:**
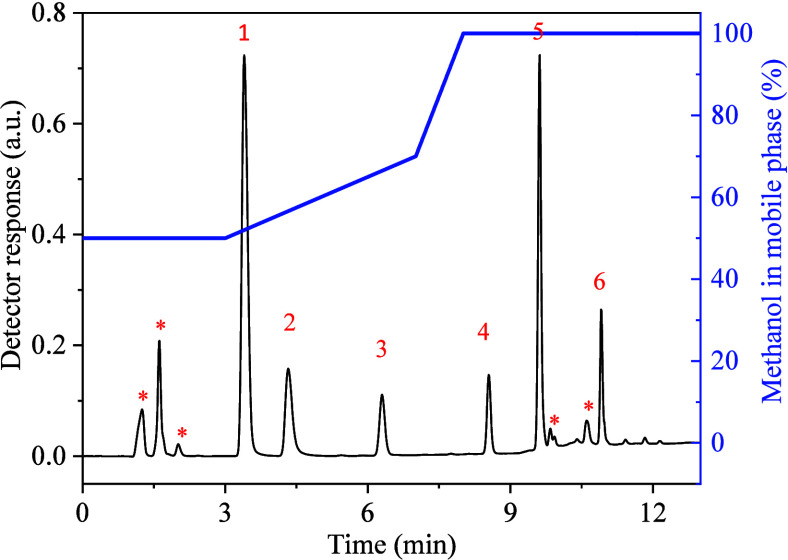
Separation of pesticide mixture extracted from a pequi pulp portion test fortified with 0.1 mg kg^–1^ using the optimized SLE-LTP method. Chromatographic conditions are listed in the experimental section. UV detection was performed at 254 nm. Peak identification: *- matrix coextractives, 1- CBDZ, 2- THIA, 3- CARB, 4- DIUR, 5- TERB, and 6- CHLO.

A validation study, conducted by European Commission guidelines and Codex Alimentarius standards, was performed to evaluate the analytical performance of the method for determining pesticide residues in pequi pulp.^27,36^ The selectivity was assessed by injecting a standard pesticide mixture prepared in matrix-matched extraction by the optimized SLE-LTP method (Figure 4). According to the chromatogram, no interference occurred in the retention times of the pesticide peaks. Therefore, their coextractives did not significantly affect pesticide residue quantification in pequi pulp.

**Figure 5:**
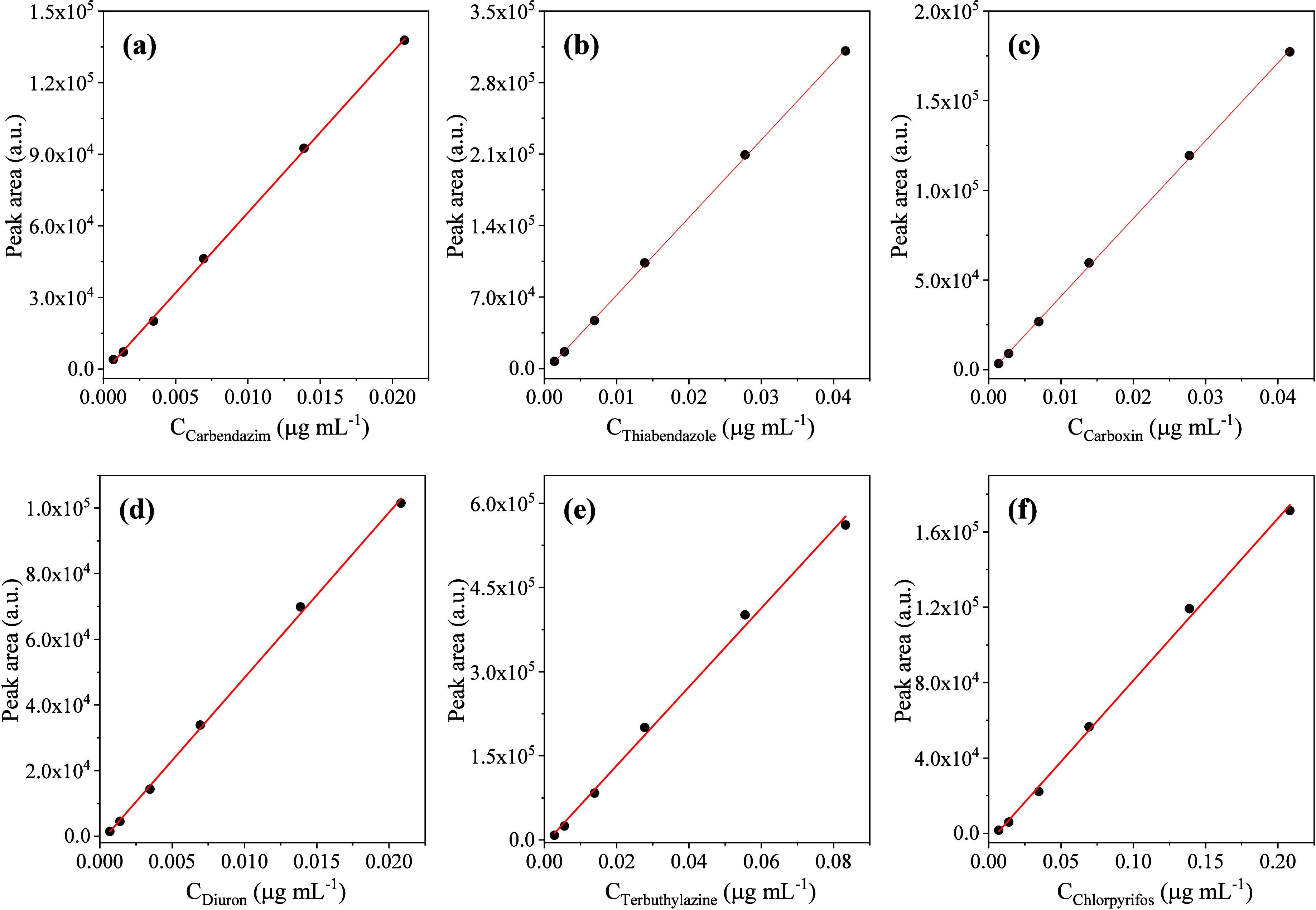
Analytical curves obtained by matrix-matched calibration for the pesticides in pequi pulp by the SLE-LTP-HPLC-PDA method: (a) Carbendazim, (b) thiabendazole, (c) carboxin, (d) diuron, (e) terbuthylazine, and (f) chlorpyrifos.

The retention time of the compounds in the chromatographic separation is directly associated with their polarity and their interaction with the C_18_ column, and the elution order is related to the reduction in the polarity (increase in LogP, Table 1) of the compounds. The linearity, range, and matrix effect of the SLE-LTP method were obtained from the analytical curves of the pesticide solvent standards and matrix-matched standards. The results for these analytical parameters, as shown in Table 8, meet the acceptability criteria of both European and Codex Alimentarius guidelines,^27,36^ where the linearity has a coefficient of regression higher than 0.99 and the matrix effect is less than 20% for all pesticides. Representative analytical curves for the studied pesticides are listed in Figure 5. The linear range was chosen from the limit of quantification to approximately 30 × LOQ, where pesticide residues, if present, are expected to be detected in the samples.

**Table 8:** Analytical Parameters of the Method for Determining Pesticide Residues in the Pequi Pulp Test Portions Using HPLC-PDA Analyses

pesticide	range (μg kg^–1^)	linearity (*R*^2^)	LOQ (μg kg^–1^)^a^	recovery (%)	RSD (%)
CBDZ	0.7–10.4	0.996	0.7	99	3
THIA	1.4–20.8	0.995	1.4	98	3
CARB	1.4–20.8	0.997	1.4	101	5
DIUR	1.4–20.8	0.998	1.4	98	5
TERB	2.8–83.3	0.998	2.8	99	6
CHLO	6.9–208.3	0.999	6.9	94	9

a Samples were fortified at the level of the instrumental limit of quantification (LOQ) (*n* = 6).

The low matrix effect values obtained for pesticides were considered a significant result for the method, as pequi pulp is a complex matrix and no additional cleanup step was used. This condition is a primary advantage of the LTP approach, which retains most of the sample’s solid impurities and lipid components in the frozen aqueous phase, thereby functioning as an extract purification step. Additionally, the method was robust throughout the validation study, as the pesticide recoveries, based on variations in extraction conditions, consistently fell within a range of 70 to 120%.

The limits of quantification (LOQ) of the method for all pesticide residues in the pequi pulp was 0.7–6.9 μg kg^–1^. These LOQ values were achieved after evaporating ethyl acetate from 4 mL of the extract and resuspending the residue in 0.2 mL of acetonitrile. Since there is no maximum residual limit (MRL) for pequi in Brazilian legislation or the Codex Alimentarius, a concentration of 10 μg kg^–1^ is considered a tolerable limit for pesticide residues in monitored food.^37^ Besides that, considering the MRL range for the studied pesticide residues in other fruits, the proposed method based on SLE-LTP can be efficiently used for this determination.^37^

The method precision (RSD) and accuracy (recovery) were determined using the optimized SLE-LTP for the six pesticides from fortified pequi pulp test portions at three concentration levels: 1 × LOQ, 2 × LOQ, and 4 × LOQ. Precision was defined in terms of repeatability and within-lab reproducibility, as recommended by the SANTE/11312/2021 document.^27^ Within-lab reproducibility was determined from the SLE-LTP of the pequi pulp fortified with the pesticides on two different days. The accuracy and precision results of the method are presented in Table 9.

**Table 9:** Accuracy (Recovery) and Precision (Repeatability and Within-Lab Reproducibility) of the SLE-LTP and HPLC-PDA Methods for Determining Pesticide Residues in Pequi Pulp (*n* = 6)^a^

	repeatability (% RSD)			within-lab reproducibility (% RSD)			recovery (%)		
pesticides	F1	F2	F3	F1	F2	F3	F1	F2	F3
CBDZ	4	7	7	13	12	7	101	99	100
THIA	6	10	6	9	10	3	98	98	103
CARB	12	9	5	11	9	7	102	101	102
DIUR	7	8	8	13	11	6	108	98	99
TERB	12	8	4	16	10	6	106	99	94
CHLO	9	7	6	14	15	14	95	94	87

a F1: samples fortified with 1 × LOQ; F2: samples fortified with 2 × LOQ; and F3: samples fortified with 4 × LOQ.

The recoveries obtained for the pesticides in pequi pulp samples using the optimized SLE-LTP method ranged from 90 to 110% at different sample fortification levels, as determined by matrix-matched calibration curves, indicating the proposed method’s good accuracy. The method precision was satisfactory, as the RSD ranged from 3 to 16%, which is below the 20% acceptable criterion at the studied concentration levels.^27^ Despite the high lipid content of pequi pulp and the complexity of its chemical composition,^38^ the SLE-LTP method, performed in a one-pot since the addition of the test portion to the extract collection, without additional cleanup steps, is efficient for extracting pesticide residues from pequi pulp with accuracy and precision. These results highlight the advantages of the LTP technique over the QuEChERS method, which involves several steps and requires an additional cleaning step for fatty samples, typically using dispersive solid-phase extraction (dSPE) with sorbents such as C_18_ (octadecylsilane), PSA (primary and secondary amines), or a mixture of both.^39,40^

### 3.3 Method Application

Five pequi samples from different areas of the Brazilian Cerrado region were analyzed by using the validated SLE-LTP-HPLC-PDA method. The proposed method did not detect any residues of the studied pesticides in any pequi samples even though they were collected from an intense agricultural area. Some possibilities for the nonoccurrence of pequi contamination are associated with the systemic application of certain active ingredients, including carbendazim, thiabendazole, and carboxin, to the seeds of crops grown in the region. So, the possibility of these compounds reaching the native forest and contaminating the pequi fruits is very low. Furthermore, the rigid structure of the pequi fruit, with a thick peel rich in lipids, proteins, carbohydrates, and fibers, can act as a chemical barrier to the permeation of contaminant molecules to the pulp, which is consumed as food.

To verify the accuracy of the proposed analytical method, the samples were fortified with 10 μg kg^–1^ of the standard pesticide mixture solution and extracted using optimized and validated SLE-LTP, as shown in Table 10. This fortification level corresponds to the tolerable limit for pesticide residues in nonmonitored food, as specified by Codex Alimentarius.^37^ The pesticide recoveries were close to 100%, confirming the efficacy of the proposed method for determining carbendazim, chlorpyrifos, terbuthylazine, thiabendazole, diuron, and carboxin in pequi pulp samples.

**Table 10:** Pesticide Recoveries in Spiked (10 μg kg^–1^) Pequi Pulp Samples Collected from Regions of Intense Agricultural Activity Using the Validated SPE-LTP Method (*n* = 3)

	recovery ± s (μg kg^–1^)					
pesticide	blank sample	sample #1	sample #2	sample #3	sample #4	sample #5
CBDZ		9.8 ± 0.4	9.8 ± 0.2	9.5 ± 0.5	8.9 ± 0.2	9.3 ± 0.4
THIA		10.8 ± 0.5	11.0 ± 0.2	9.7 ± 0.9	9.7 ± 0.1	9.6 ± 0.2
CARB		10.1 ± 0.8	9.9 ± 0.4	9.1 ± 1.0	10.1 ± 0.1	9.4 ± 0.3
DIUR		9.9 ± 0.3	10.4 ± 0.4	9.7 ± 0.3	8.8 ± 0.4	10.0 ± 0.5
TERB		10.5 ± 0.8	10.9 ± 0.3	10.6 ± 0.6	9.8 ± 0.8	10.1 ± 0.4
CHLO		11.0 ± 0.1	11.0 ± 0.4	10.2 ± 0.8	10.3 ± 0.4	10.0 ± 0.4

## 4 Conclusions

In this work, an extraction method (SLE-LTP) of pesticide residues in pequi pulp was developed and optimized, with analysis by high-performance liquid chromatography and diode array detection, which showed good accuracy and precision in the determination of residues of the pesticides carbendazim, chlorpyrifos, terbuthylazine, thiabendazole, diuron, and carboxin from pequi pulp samples. Statistical tools enabled the optimization of eight variables in the SLE-LTP method, resulting in recoveries of nearly 100% for all six pesticides. Although the extract presented a light-yellow coloration, the results indicated that the matrix effect is negligible, without the need for an additional cleanup step, performing the extraction process from the addition of the sample and ethyl acetate inside the Falcon tube (one-pot), freezing, and removing the organic fraction directly for chromatographic analysis, without subsequent purification. Thus, the proposed method simplifies the sample preparation process in terms of steps and the analyst’s handling. The proposed method also provided an adequate LOQ for monitoring contamination by the studied pesticides in pequi as they were below the MRL values. The evaluation of pequi samples in regions of intense monoculture cultivation indicated the absence of pesticide residues.
